# AChE-based electrochemical biosensor for pesticide detection in vegetable oils: matrix effects and synergistic inhibition of the immobilized enzyme

**DOI:** 10.1007/s00216-022-04448-y

**Published:** 2022-11-29

**Authors:** Dimitra Tsounidi, Dionysios Soulis, Fotini Manoli, Apostolos Klinakis, George Tsekenis

**Affiliations:** 1 Biomedical Research Foundation of the Academy of Athens, Athens, Greece; 2 Minerva SA Edible Oils & Food Enterprises, Athens, Greece

**Keywords:** Acetylcholinesterase, Electrochemical biosensor, Pesticide, Fatty acids, Vegetable oils, Enzyme inhibition

## Abstract

Enzyme-based electrochemical biosensors have been widely deployed for the detection of a range of contaminants in different food products due to their significant advantages over other (bio)sensing techniques. Nevertheless, their performance is greatly affected by the sample matrix itself or by the matrix they are presented with in pretreated samples, both of which can impact the accuracy as well as the sensitivity of the measurements. Therefore, and in order to acquire reliable and accurate measurements, matrix effects and their influence on sensor performance should be taken into consideration. Herein, acetylcholinesterase (AChE)-modified electrochemical sensors were employed for the detection of pesticides in vegetable oils. Sensor interrogation with pretreated oil samples, spiked with carbofuran, revealed the inhibitory potential of the extracted matrix varies between different types of vegetable oil and their fatty acid content. In addition, synergies between the extracted matrix from different types of vegetable oils and the carbamate pesticide, carbofuran, were observed, which led to significant deviations of the sensor’s performance from its anticipated behavior in buffered solution. Taking the aforementioned into consideration, appropriate calibration curves for each type of vegetable oil were drafted, which allowed for the highly reproducible determination of different pesticide concentrations in pretreated real samples. Collectively, a better understanding of AChE inhibition by single or multiple contaminants present in vegetable oils was gained, which can find many applications in numerous fields, ranging from sensor development to the design of new pesticides and medicinal products.

**Supplementary Information:**

The online version contains supplementary material available at 10.1007/s00216-022-04448-y.

## Introduction

Cholinesterases, AChE and butyrylcholinesterase (BuChE), and their inhibition by a diverse set of biogenic (for example, glycoalkaloids, aflatoxins, and anatoxins) [1] as well as synthetic compounds (such as pesticides and nerve agents) and even heavy metals [2] have been the subject of intense research due to their numerous applications, ranging from the design of therapeutic agents for the treatment of neurodegenerative disorders [3] to the fabrication of biosensors for the detection of analytes of clinical significance, but also contaminants or toxins relevant to the food and military industries as well as the environment [4, 5]. As far as the latter are concerned, a bewildering number of biosensing platforms have been proposed in the past 60 years, since the first biosensor based on AChE inhibition was developed, the majority of which targets organophosphate and carbamate pesticide detection in food and environmental samples [4, 6, 7]. Their operation is based on different signal transduction principles, including electrochemical [8–10], optical [11], photoelectrochemical [12, 13], and piezoelectric [14]. More recently, the development of novel materials such as liquid crystals [15], metal organic frameworks [16], nanozymes [17], and quantum dots [18] has broadened the detection schemes that can be realized to include, for example, colorimetry [19] and fluorescence [20], which, along with the utilization of microfluidics and paper-based devices [21–23], have opened up new possibilities in the field of pesticide detection, attesting to the potential of cholinesterase-based sensors in achieving the fast and reliable quantification of these omnipresent and persistent contaminants.

Among the different signal transduction principles, detection of pesticide-induced cholinesterase inhibition with the use of electrochemistry still dominates the field, due to the significant advantages it possesses over other sensing principles [24]. Electrochemical sensors make the most of the enzyme’s inherent catalytic activity and the electrochemically active nature of the catalysis product, allowing highly sensitive measurements to be acquired without the need for multiple and complex sensor surface functionalization chemistries nor the use of labels [25]. Pesticide quantification can thus be achieved directly, with a relatively simple sensor design and at a low cost, all of which can facilitate the deployment of cholinesterase-based sensors in in the field, at the point of need [26]. In addition, the chemical modifications implemented on the sensor surface help address one of the main drawbacks of enzyme-based sensors, namely instability, permitting robust assays to be developed that can withstand prolonged storage with minimal loss of operational activity, thus paving the way for the commercialization of these sensors [27].

As far as the performance of electrochemical cholinesterase-based sensors is concerned, the main research focus has been on improving their sensitivity and selectivity, either through the employment of genetically modified enzymes or through the modification of the transducer with electrochemical mediators [28]. Recent advances in nanotechnology and material science have allowed the development of sensors of unparalleled sensitivity that exploit the structural and functional properties of carbon and metallic nanomaterials as well as composites thereof with conducting polymers, thus significantly enhancing sensor performance and storage stability [29–31]. By contrast, real sample analysis has been frequently overlooked, as a sensor’s sensitivity and specificity are usually reported on the basis of its interrogation and optimization with buffered solutions. Nonetheless, the instrumental limit of detection obtained with the use of “laboratory” solutions can be quite different to the method’s limit of detection obtained when a developed sensor is put to the test with real samples [32]. To determine the latter, the analyte must be measured in the actual matrix of the intended use, which is far more complex than a buffered solution and its impact can be manifested in several ways, such as through the interaction of the analyte with molecules in the matrix and its adverse effect on the sensor’s limit of detection. Matrix interference and real sample analysis are common challenges to all biosensors, with various strategies having been devised to overcome them, depending on the nature of the sample and the biorecognition molecule [33]. In water samples, for example, the effect of the matrix can be prevented by diluting and/or buffering the sample as well as adjusting its pH, while in the case of antibody-based sensors, addition of detergents or immolation proteins is a routine practice [34]. Cholinesterase-based sensors, in specific, are highly susceptible to false signals when interrogated with samples whose matrix is either unknown or variable, such as the matrix presented by environmental or food samples that may contain one or more of the numerous compounds known to interfere with the enzyme’s catalytic activity [35]. These include alkaloid and non-alkaloid natural compounds such as terpenoids, not only coumarins but also phenols, which are omnipresent in food and environmental samples [36]. In addition, a sample’s fat or alcohol content can induce enzyme inhibition, while interference with the enzyme’s catalytic activity has been unexpectedly shown even with river or seawater samples, whose pH and salt concentration were not controlled [37].

To address the need of removal of the aforementioned interferents or at least to mitigate their negative impact on the activity of the enzyme, sample pretreatment is usually undertaken [35]. Sample pretreatment protocols differ considerably and depend on both the analyte to be extracted and the type of food or environmental sample, while they commonly involve the use of organic solvents, known for their inhibitory effect on cholinesterases [38]. In the case of cholinesterase-based biosensors, therefore, and despite the substantially “cleaner” sample obtained upon sample pretreatment, sensor performance deteriorates due to the presence of the organic solvent. To further aggravate matters, small quantities of lipids, proteins, or other organic molecules persist in the extracted solution, which can lead to further reduction of sensor sensitivity, specificity, and measurement reliability. It becomes apparent, therefore, that matrix interference of either a real or of an extracted sample with the sensor performance can be disproportionately large, resulting in large deviations from the anticipated behavior. Hence, calibration of the developed enzyme-based sensors in matrices relevant to the real sensor application is of paramount importance. In this way, the causative reason behind aberrations in the measurements can be identified and accounted for, whether, for example, it is due to the low recovery of the analyte following sample pretreatment, or due to matrix effects and the presence of interferents, or even a combination thereof, ultimately allowing for the development of truly robust assays and of cholinesterase-based biosensors capable of acquiring reliable readings.

Despite the significance of evaluating sensor response and performance with real or pretreated samples, the number of studies on cholinesterase-based sensors for pesticide detection in environmental and food samples remains limited, while even fewer embark on a detailed validation of the developed sensors against standard laboratory techniques, such as chromatography [39, 40]. Real sample analysis with the use of said sensors has been undertaken in water, milk, fruits, vegetables, pulses, grains, and oil samples and has been extensively reviewed elsewhere [24, 41]. Interestingly, no major matrix interference, other than the effect of the organic solvent itself, and analyte recoveries close to 100% were described in all of the studies involving the analysis of pesticide-spiked real samples, with the exception of milk and oil samples. In the former, it was found that sensor response deviations increase with increasing fat content of milk [42], while in the latter, a more complex relationship between sensor response and the targeted pesticide and extraction protocol was identified. Similarly, in our recently published work [43], increased inhibition of the immobilized enzyme was recorded upon interrogation of the fabricated sensor with pretreated samples of olive oil spiked with pesticides relative to the effect of identical concentrations of pesticides in buffered solutions and despite having accounted for and already subtracted the inhibitory effect of the extracted matrix.

Herein, in an attempt to gain a better understanding of cholinesterase inhibition, an extensive investigation into the possible synergistic effects of pesticides and fatty acids present in extracted oil samples was undertaken. The investigation was extended to different types of vegetable oils, to account for the effect vegetable oil composition and fatty acid content have on the observed deviations of the sensor performance. The fabricated electrochemical sensors were interrogated both with samples extracted from oils spiked with a range of pesticide concentrations and with samples prepared by spiking a common pre-extracted matrix. This allowed, for the first time and to the best of our knowledge, the interference of the extracted matrix from different types of vegetable oil with the enzyme activity to be evaluated, which along with the synergistic inhibition exerted by pesticides were taken into consideration while drafting new calibration curves that reflect more accurately the anticipated sensor response in the analysis of real samples.

## Materials and methods

### Materials

Acetylcholinesterase (AChE from electric eel), acetylthiocholine iodide (ATChI) and carbofuran (PESTANAL®, analytical standard), low molecular weight chitosan (CS), glutaraldehyde, Nafion™ (perfluorinated ion-exchange resin, 5% v/v solution in lower alcohols/water), bovine serum albumin (BSA), and PTFE Whatman syringe filters with a pore size of 0.45 μm were purchased from Sigma-Aldrich (St. Louis, MO, USA). Conductive carbon black (CB) (Vulcan XC 72R) with average particle size of 50 nm and typical bulk density of 6 lbs/ft^3^ was kingly supplied from Cabot Corporation’s representatives in Greece (RAWCHEM). Sodium phosphate dibasic, potassium phosphate monobasic, potassium chloride, potassium iodide, and sodium hydroxide were obtained from Honeywell Fluka™ (North Carolina, USA). Acetonitrile (ACN) and hydrochloric acid (HCl) were purchased from Fisher Scientific (Bishop Meadow Road, Loughborough, UK). All solutions were prepared with Milli-Q water (18.2 MΩ∙cm).

### Apparatus

Cyclic voltammetry (CV) and chronoamperometric (CA) measurements were performed using disposable carbon screen-printed electrodes (SPEs) (Carbon DRP-110) consisting of carbon ink based working electrode with 4 mm diameter, a carbon counter electrode, and an Ag reference electrode, along with a cable connector for screen-printed electrodes (DRP-CAC4MMH), which were purchased from Metrohm DropSens (Oviedo, Asturias, Spain). A BioLogic SP-200 potentiostat was used for all electrochemical measurements (BioLogic, Seyssinet-Periset, France).

### Preparation of calibrators and oil samples

Stock solution of carbofuran was prepared (1 g/L in ACN), which was used both for spiking vegetable oil samples, extracted vegetable oil matrices, and to prepare the pesticide calibrators in buffered solution. Solutions of different concentrations of carbofuran were prepared in the aforementioned. The buffer solution used was 0.05 M phosphate buffer, 0.1 M KCl, and pH 7.4. In all cases, the final concentration of ACN in the samples interrogated was 5%. Different types of vegetable oil were provided by MINERVA S.A, Greece, which had previously been analyzed by applying a standard technique of Oils and Fats Accredited LC-QTOF∙GC–MS/MS using the modified method of analysis with code number O.B.05.48 (EN 15,662:2018 and SANTE/11813/2017 of the European Commission) and were certified as pesticide-free. Moreover, the percentage composition of fatty acids in each vegetable oil sample was determined by MINERVA S.A. by gas–liquid chromatography using a flame-ionization detector (FID) for the analysis of fatty acid methyl esters (Supplementary Information). For the preparation of the spiked and subsequently extracted pesticide samples, 10 g of vegetable oil was mixed with 10 mL of ACN and vigorously agitated for 5 min to form an emulsion. The latter was left undisturbed for 15 min, so that the two phases separate. Subsequently, 1 mL of the supernatant was obtained using a syringe and filtered through a PTFE/L 0.45 μm syringe filter. The filtrate was mixed with phosphate buffer (1:20 ratio). Similarly, for the samples of pesticide spiked directly in extracted vegetable oil, the different vegetable oil samples were extracted as previously and different pesticide concentrations were spiked into them always maintaining an ACN/buffer ratio of 1:20.

### Fabrication of AChE-based biosensor

For the fabrication of working electrode surface with carbon black, a CB/CS solution was firstly prepared following the protocol previously presented in [43]. Briefly, an aqueous solution of 0.05 M HCl was prepared and heated at 90 °C. Sufficient CS was added and dissolved under stirring for approximately 30 min, resulting in a final concentration of 0.05% w/v. Thereafter, 3 mg/mL of CB was added to the CS solution, which was subsequently allowed to cool to room temperature. The mixture was left overnight under stirring in order to achieve the best possible dispersion. Prior to its usage, the CB/CS solution was vigorously vortexed. The enzyme was either mixed in the CB/CS dispersion (one-step procedure) or was covalently immobilized onto the CS/CB-modified SPEs (multi-step procedure). For the former, the enzyme was diluted in phosphate buffer and added in the CB/CS dispersion in a 1:2 v/v ratio to a final concentration of 23 μg/mL. For the multi-step modification of the electrodes, 3 μL οf the CB/CS mix was drop-casted onto the working electrode (2 sequential additions) and left to dry prior to the application of 2 μL of glutaraldehyde (0.25% v/v in water). Finally, AChE was allowed to bind covalently onto the electrode following application of 2 μL of the enzyme mixture (3% BSA, 0.1% Nafion, AChE dissolved in PBS buffer in equal ratios by volume) onto the glutaraldehyde-modified CB/CS-functionalized SPEs. The final concentration of the enzyme used was 75 μg/mL unless otherwise stated.

### Electrochemical determination of carbofuran

The inhibition of AChE by carbofuran was evaluated and electrochemically, by monitoring the current generated by the enzymatic oxidation of the pseudosubstrate thiocholine (ATChI) with the use of the CB/CS/AChE-modified SPEs, as described in our previous published work [43]. The percentage inhibition (*I*%) was calculated using the following formula:

1 $$I\%=(({I}_{0}-{I}_{1})/{I}_{0})\times 100$$

where *I*_0_ is the activity of the uninhibited enzyme and *I*_1_ that of the enzyme upon incubation with different concentrations of carbofuran. For the electrochemical determination of the percentage inhibitions of carbofuran exerted onto the enzyme, the current intensity at the CB/CS/AChE-modified SPEs was recorded at a constant applied voltage of + 0.45 mV unless otherwise stated. Following an initial voltage application for 5 min in phosphate buffer solution to stabilize the electrode, pesticide detection was achieved in a three-step procedure as follows: first, the initial response of the electrode to 2.5 mM ATChI (60 μL) was recorded (baseline); then the electrode was incubated in a solution (buffered or pretreated sample) containing a known concentration of pesticide for 20 min; and finally, the residual response of the electrode was recorded again. To account for the inhibition to the enzyme activity caused by acetonitrile present in the pesticide solutions, 5% v/v ACN was added in all of the used solutions (buffer for stabilization, substrate dissolved in buffer). The measured signal corresponded to the difference of current intensity between the baseline and that measured upon incubation with the pesticide solutions.

## Results and discussion

### Assay optimization

**Fig. 1:**
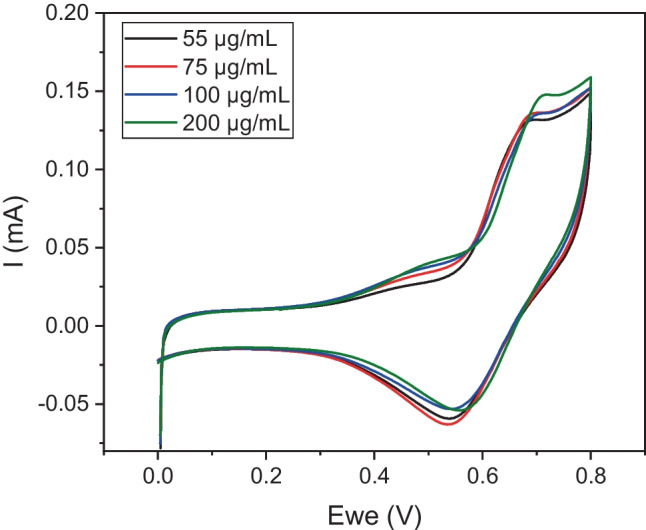
Cyclic voltammograms obtained for CB/CS-modified carbon SPEs using the multi-step approach with different concentrations of AChE immobilized and interrogated 3 min upon incubation with 2.5 mM ATChI in buffer solution, at a scan rate of 50 mV/s

Prior to testing the different extracted or spiked vegetable oil samples, we attempted to acquire a calibration curve of the percentage inhibition of AChE by using different concentrations of carbofuran and employing the optimized protocol presented in [43]. Nevertheless, no significant differences between the current intensities recorded in the absence or in the presence of the pesticide at an applied potential of + 0.25 mV were recorded. In addition, the recorded current intensities following both approaches of fabricating the biosensors were lower than those previously acquired. As the enzyme and screen-printed electrodes employed were different (new batch) to the ones employed in our previous publication, we hypothesized that differences in sensor performance could be due to the consumables and reagents used. This is why the sensor performance was evaluated and optimized anew. Interrogation of the CB/chitosan (CS)-modified electrodes by CV fabricated using the multi-step approach and following the immobilization of different concentrations of the enzyme reveals that overpotentials exceeding + 0.4 need to be applied for thiocholine oxidation, while the largest differences in the recorded current intensities were recorded at a potential of + 0.45 V (Fig. 1). This value differs significantly from the potential required for thiocholine oxidation in our previous published work and could be explained taking into consideration differences in the new batch of SPEs employed [43]. Using this applied potential and chronoamperometry, the apparent Km for the sensors fabricated following the one-step and multi-step approaches was compared. The results presented in Fig. S1 reveal that the enzyme immobilized via glutaraldehyde (multi-step approach) onto the electrode surfaces has roughly ten times less affinity for its substrate (Km = 1.5 mM) than the enzyme entrapped in the CS matrix (Km = 0.15 mM), which is in agreement with the results obtained in our previous study. However, the current signal obtained for the one-step enzyme immobilization approach was quite low, which once again could be attributed to differences in the SPEs. The current intensity values obtained following the one-step approach would not allow chronoamperometric measurements with high resolution and that is why the multi-step approach was used in all subsequent investigations presented in the manuscript.

**Fig. 2:**
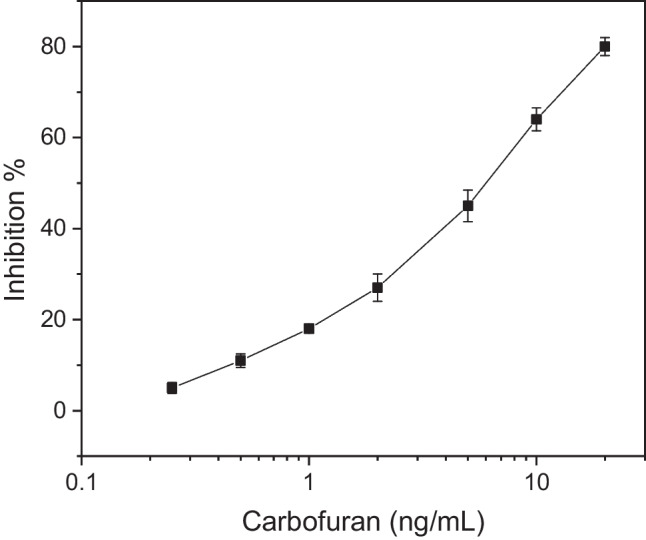
Percentage inhibition of the AChE obtained after incubation with carbofuran’s calibrators prepared in assay buffer. Each point is the mean value of three measurements ± SD

Following protocol optimization, a calibration curve of the percentage inhibition of AChE with increasing concentrations of carbofuran in assay buffer was obtained (Fig. 2). It is worth stressing out that the concentrations of pesticide shown in the diagram correspond to the diluted concentrations following addition of pesticide in ACN at a 1:20 ratio to solution buffer. The concentration of pesticides in the undiluted calibrator solutions was 20 times higher. The biosensor could effectively therefore detect carbofuran with a limit of detection equal to 0.1 ng/mL (4.5 × 10^−10^ M), which translates to 2 ng/mL (0.9 × 10^−9^ M) in the actual calibrator sample. The linear dynamic range achieved extended from 0.2 to 20 ng/mL (9 × 10^−10^ M–9 × 10^−8^ M), thus from 4 up to 400 ng/mL (1.8 × 10^−8^ M–1.8 × 10^−6^ M) in the actual samples. It appears that with the optimized protocol, a similar limit of detection to that previously reported was achieved while the linear dynamic range was extended by an order of magnitude.

### Sample matrix effect

**Fig. 3:**
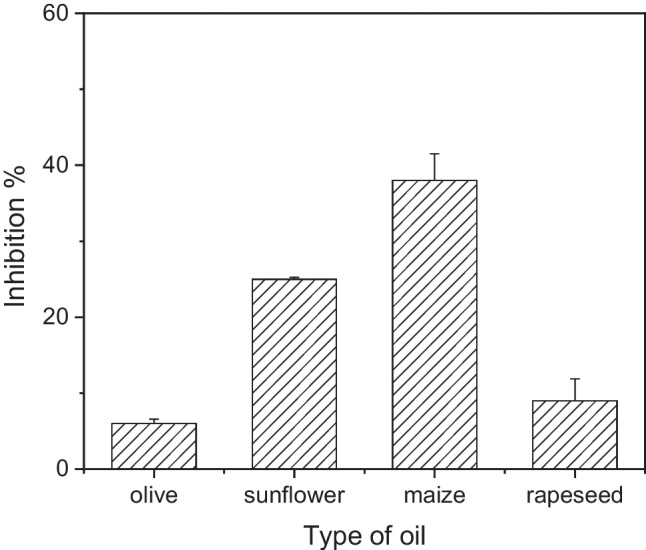
Percentage inhibition of AChE-modified biosensors obtained following incubation with extracted matrices from olive, sunflower, maize, and rapeseed oil in the absence of any pesticide. Each point is the mean value of five measurements ± SD

Following the acquisition of the new calibration curve for carbofuran in buffer solution and the optimization of the assay conditions, we then proceeded to the assessment of the effect the extracted matrix had on the immobilized enzyme. For this purpose, four different types of vegetable oils were employed. All of them had already been tested for the presence of any pesticides with the use of an established analytical method. Sample extraction was achieved as described in the “Materials and methods” section. Extracted samples were mixed at 1:20 ratio with buffer and incubated with the enzyme-modified sensors. The results obtained clearly demonstrate that the extracted matrix has a significant impact on the activity of AChE, which is highly dependent on the type of vegetable oil (Fig. 3).

The recorded inhibition could be explained taking into account that there are fatty acids still present in the extracted matrix, which has a pale-yellow hue. AChE inhibition has been extensively studied due to its implications to human health and the design of pharmaceutical compounds as well as its importance in the design of new insecticides [44]. AChE inhibitors can be divided between those that bind the bottom of the gorge that forms the enzyme’s active site and those that bind to the “peripheral anionic site” (PAS), which is remote from the catalytic site. Hydrophobic compounds like the fatty acids present in the extracted matrices have been found to penetrate into the gorge; nevertheless, and unlike the fatty acids, these compounds are mostly polycyclic. A possible explanation of AChE’s inhibition by fatty acids has been provided by Loesche et al. [45]. They have shown that α,β-unsaturated fatty could fit very well into the AChE hydrophobic binding pocket that acts as the active site, while the carboxyl groups of the compounds fit reasonably into the PAS. This group has undertaken an extensive investigation into the effect different saturated and unsaturated fatty acids have on AChE’s activity and they have concluded that the most potent inhibitors are those fatty acids made up of 14 or 16 carbons, irrespectively of their degree of saturation. Research carried out on oils extracted from fish, plant oils, and plant pulps has shown that oils with high poly-unsaturated fatty acid content are the most potent AChE inhibitors or unsaturated fatty acids (UFA) in general, with emphasis on oleic and linoleic acids, also show high percentage of enzyme inhibition [46–49].

Taking this information into account alongside the fatty acid composition of the different vegetable oils employed in this study (Table 1), the higher inhibition on AChE exerted by sunflower and maize oil can be explained by their higher poly-unsaturated fatty acid (PUFA) content, which can be accounted for by the significantly higher percentage of poly-unsaturated linoleic acid. Further to this, palmitic acid is also found in higher percentage concentrations in both sunflower and maize oil and could also contribute to the higher impact these vegetable oils have on the activity of the enzyme. However, research has shown that palmitic acid has a low inhibitory potential on AChE [50]. The same group has also shown that linolenic acid is a potent inhibitor of AChE, which does not correlate with the results obtained for the vegetable oils examined in this study, where sunflower oil actually had the lowest content of this poly-unsaturated fatty acid. The latter illustrates that identifying a single fatty acid responsible for the higher percentage inhibition of AChE exerted by the extracted matrix of sunflower and maize oil is not an easy task, especially taking into consideration the complex fatty acid profile of vegetable oils.

**Table 1:** Fatty acids composition of the different samples of vegetable oils employed in this study. Olive oil samples from three different geographic origins, as well as sunflower and maize oil samples from two different geographic locations, were analyzed

Fatty acids (%)	Olive oil (1)	Olive oil (2)	Olive oil (3)	Sunflower oil (1)	Sunflower oil (2)	Maize oil (1)	Maize oil (2)	Rapeseed oil
Lauric (C12:0)	0.01	0.01	0.01		0.11		0.02	0.03
Myristic (C14:0)	11.68	12.04	11.50	0.10	0.09	0.03	0.04	0.06
Palmitic (C16:0)	0.91	0.92	0.93	7.18	7.06	11.76	11.76	4.84
Palmitoleic (C16:1)	0.05	0.04	0.04	0.19	0.22	0.19	0.15	0.26
Stearic (C18:0)	2.59	2.70	2.53	3.28	3.28	1.82	1.80	1.71
Oleic (C18:1)	77.62	76.40	76.83	36.04	40.08	29.63	30.25	62.38
Linoleic (C18:2)	5.36	6.09	5.72	51.69	47.78	54.17	53.95	20.13
Linolenic (C18:3)	0.71	0.73	0.73	0.07	0.06	1.16	0.99	8.00
Arachidic (C20:0)	0.48	0.48	0.47	0.26	0.27	0.40	0.42	0.54
Eicosenoic (C20:1)	0.32	0.30	0.31	0.14	0.16	0.26	0.28	1.16
Behenic (C22:0)	0.17	0.16	0.16	0.65	0.72	0.12	0.13	0.32
Lignoceric (C24:0)	0.01	0.01	0.01	0.26	0.22	0.15	-	0.12
Trans-oleic	0.01	0.02	0.02	-	-	-	-	-
Trans-linoleic	0.02	0.02	0.03	-	-	-	-	-
Total MUFA^a^	78	76.76	77.2	36.37	40.46	30.08	30.68	63.8
Total PUFA^b^	6.57	7.32	6.95	52.02	48.11	55.73	55.36	28.67
Total SFA^c^	15.37	15.84	15.14	11.47	11.48	13.88	14.03	7.08

^a^*MUFA*, monounsaturated fatty acids; ^b^*PUFA*, poly-unsaturated fatty acids; ^c^*SFA*, saturated fatty acids

### Detection of carbofuran in vegetable oil samples

**Fig. 4:**
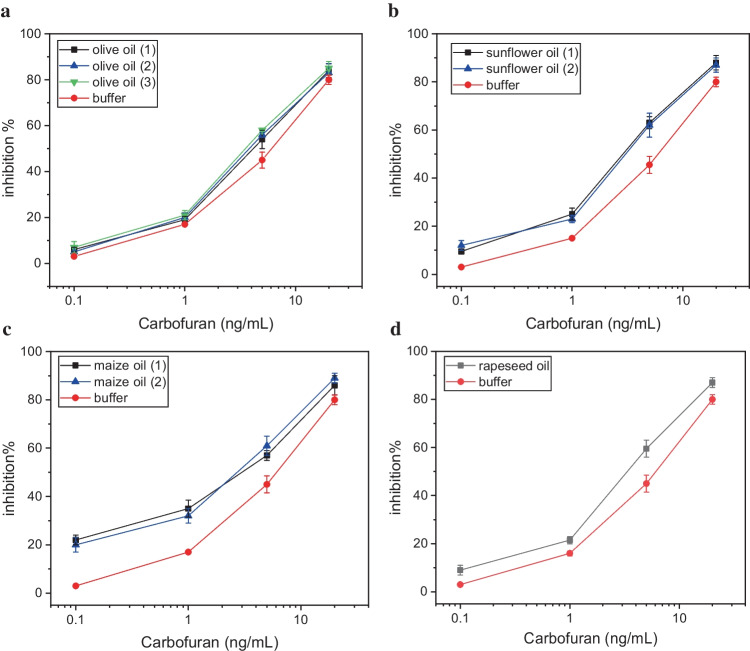
Percentage inhibition of the AChE-modified biosensors obtained after incubation with calibrators of carbofuran prepared in assay buffer (red line in all of the graphs) and extracted matrix from **a** olive, **b** sunflower, **c** maize, and **d** rapeseed oil of different geographic origins. Each point is the mean value of three measurements ± SD. #

Having estimated the percentage inhibition due to the effect of the extracted matrix of the different types of vegetable oils, measurements for the determination of carbofuran were conducted. Different concentrations of the pesticide were spiked into the extracted matrix of the four different types of vegetable oils. The percentage inhibition of AChE due to the presence of carbofuran was estimated taking into account the inhibition of the matrix itself of each oil sample (Fig. 3). The calibration curves obtained for the different oil samples depict the additional inhibition of AChE caused by increasing carbofuran concentrations, compared with the inhibition of the enzyme obtained with samples of the same concentration of pesticide in buffered solution (Fig. 4). In all of the examined samples, carbofuran can be detected over the same linear dynamic range as in assay buffer, with little difference observed between samples of the same type of vegetable oil but of different geographic origin, indicating that the differences in percentage inhibition are due to the samples’ composition and their fatty acid content as already discussed. Moreover, the sensitivity of the proposed biosensor did not get affected and carbofuran could be detected at concentrations below the MRLs of European Regulation (10 ng/mL, EU 396/2005).

Interestingly, and despite the fact that the inhibition due to the extracted matrix has been accounted for, the percentage of AChE inhibition was found to be higher in spiked pretreated oil calibrators than in the calibrators of the same concentration prepared in assay buffer. It seems very plausible, therefore, that fatty acids have a synergetic effect along with the pesticide, leading to an increased inhibition on the enzyme’s activity. This additive inhibitory effect on AChE is higher in maize oil samples, and minimal in olive oil samples (Table 2). In fact, mixtures of organic insecticides of the organophosphate types or of the carbamate type, with certain fatty acids or their salts, have been found to have enhanced insecticidal activity, a discovery that was patented in the USA in 1989 (US4861762A). Therein, it was suggested that mixtures of C18 fatty acids (saturated as well as unsaturated) with carbofuran are the most potent inhibitors of AChE, which could explain the high synergistic effect of this specific pesticide in maize oil, as the latter has the highest concentration of linolenic acid, a PUFA C18 fatty acid. Another possibility is that the vegetable oils employed in this study contained pesticides at very low concentrations, which could not be detected during their analysis with the certified analytic methodology, or even low concentrations of heavy metals, both have been shown to act synergistically on acetylcholinesterase [51, 52]. This explanation seems less likely as the additive inhibition exerted by different concentrations of carbofuran is consistent among samples of the same type of vegetable oil but different geographic origins, pointing to the fact that it is the oil’s fatty acid profile that makes the difference and not an individual sample’s characteristics and pesticide or heavy metal burden.

**Table 2:** Percentage inhibition of AChE-modified biosensors obtained after incubation with calibrators of carbofuran prepared in extracted matrix from olive, sunflower, maize, and rapeseed oil

Carbofuran (ng/mL)	Inhibition % Olive oil	Inhibition % Sunflower oil	Inhibition % Maize oil	Inhibition % Rapeseed oil
0.1	7 ± 2.5	9.5 ± 0.5	22 ± 2	9 ± 2
1	21 ± 2	25 ± 2.5	35 ± 3.5	21.5 ± 1.5
5	58 ± 0.5	63 ± 2.5	57 ± 2	59.5 ± 3.5
20	85 ± 3	88 ± 3	86 ± 4	87 ± 2

**Fig. 5:**
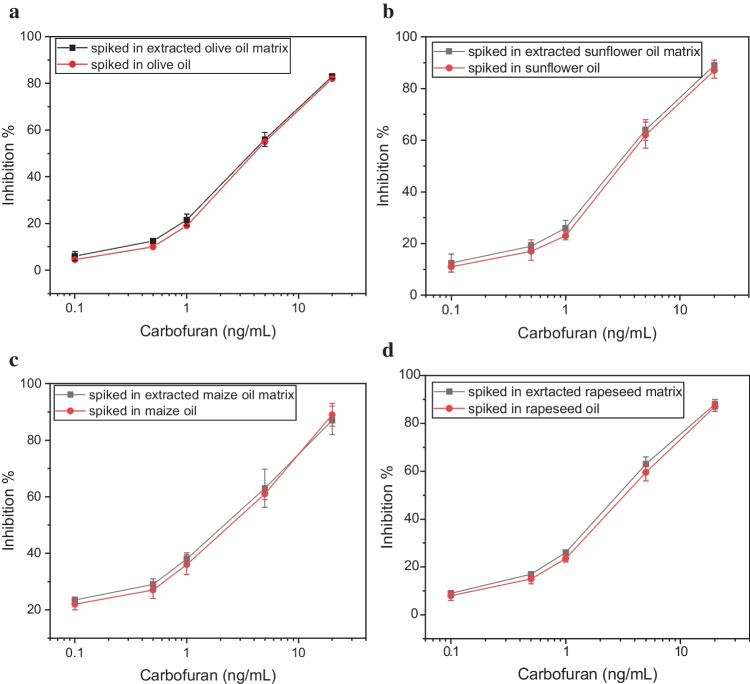
Percentage inhibition of the AChE-modified biosensors obtained following their incubation with calibrators of carbofuran spiked either in the extracted vegetable oil matrix or in the whole vegetable oil sample: **a** olive, **b** sunflower, **c** maize, and **d** rapeseed oil and then extracted. Each point is the mean value of three measurements ± SD

In addition to the investigation undertaken with calibrator samples prepared by spiking different concentrations of pesticide in the already extracted vegetable oil matrices, the same concentrations of pesticide were also spiked to the vegetable oil samples themselves and then extracted following the procedure described in the “Materials and methods” section. The obtained results validate the extraction protocol as well as the accuracy of the developed methodology (Fig. 5).

## Conclusion

The results presented in this study highlight the importance of validating the performance of a developed enzyme-based biosensor with an adequate number of real samples, to account for their heterogeneity and to record any deviations in sensor response due to interferents present in nature that would otherwise be hard to simulate in a lab environment. More specifically, it was found that the degree of enzyme inhibition varies tremendously between matrices extracted from different types of vegetable oil and depends greatly on their fatty acid content. The lowest percentage inhibition was recorded for matrices extracted from olive and rapeseed oil samples (6% and 9%, respectively), while matrix interference is significantly in the case of sunflower and oil (around 23%). Pretreated maize samples, on the other hand, almost halved the enzyme activity (40%) inhibition. Moreover, establishing a straightforward correlation between the percentage enzyme inhibition and the fatty acid content of each type of vegetable oil proved to be difficult, with unsaturated fatty acids ranking high as probable potent interferents. Nevertheless, more research has to be carried out into the complex interactions that develop between various fatty acids and AChE in order to get a better understanding of the mechanisms of enzyme inhibition in the presence of multiple interferents. Equally importantly, the synergistic effect of pesticides, such as carbofuran, and fatty acids on the inhibition of the enzyme was established. Furthermore, it was shown that the aforementioned combined inhibitory action did not have a negative impact on sensor sensitivity nor on the linear dynamic range over which different concentrations of the pesticide can be detected. In fact, the calibration curves that were drafted anew for each type of vegetable oil permit the detection of pesticide residues with increased reliability and accuracy. The insights gained can also be exploited not only in the design of new insecticide formulations that are more potent while at the same time friendlier to the environment but also in the design of novel therapeutic compounds for the treatment of neurodegenerative disorders.

## Supplementary Information

Below is the link to the electronic supplementary material.

Supplementary file1 (DOCX 67.4 kb)
