# Supplementary material for: AChE-based electrochemical biosensor for pesticide detection in vegetable oils: matrix effects and synergistic inhibition of the immobilized enzyme
Source: Anal Bioanal Chem. 2022 Nov 29;415(4):615–25. doi: 10.1007/s00216-022-04448-y (PMC9839810; doi:10.1007/s00216-022-04448-y)
Supplement: Supplementary file 1 — Supplementary file1 (DOCX 67.4 kb) [file 216_2022_4448_MOESM1_ESM.docx]

**Supplementary Information**

**AChE-based electrochemical biosensor for pesticide detection in vegetable oils: matrix effects and synergistic inhibition of the immobilized enzyme**

**Dimitra Tsounidi^1^, Dionysios Soulis^1^, Fotini Manoli^2,^ Apostolos Klinakis^1^, George Tsekenis^1^***

^1^ Biomedical Research Foundation of the Academy of Athens, Athens, Greece

^2^ Minerva SA edible oils & Food Enterprises, Athens, Greece

* Corresponding author: [gtsekenis@bioacademy.gr](mailto:gtsekenis@bioacademy.gr)

**Analysis of fatty acid methyl esters by gas chromatography**

Before analysis by Gas Chromatography (GC), a pretreatment procedure of oil samples was followed. When necessary, the samples were purified by passing the oil through a silica gel solid-phase extraction cartridge. A silica gel cartridge was placed in a vacuum elution apparatus and washed with 6 mL of hexane, without vacuum. Then a solution of the oil (0.12 g approximately) in 0.5 mL of hexane was loaded onto the column. The solution was pulled down and then eluted with 10 mL of hexane/diethyl ether (87:13 v/v). Then, the received elute was evaporated to dryness in a rotary evaporator under reduced pressure at room temperature. The residue was dissolved in 1 mL of heptane for fatty acid methyl ester preparation and analysis by GC. For the sample’s pretreatment, in a 5 mL screw-top test tube with cap fitted with a PTFE joint, 2 mL of heptane were added in 0.1 g of the oil sample, and then, 0.2 mL of methanolic potassium hydroxide solution were added, followed by vigorous shaking for 30 s. The mixture was left to stratify until the two phases were separated. The supernatant, that contained the methyl esters, was removed and the solution was ready for injection. The analysis of fatty acid methyl esters was performed through GC, employing capillary columns and a Flame-Ionisation Detector (FID). The internal diameter of the capillary column should be between 0.20 to 0.32 mm, while a length of 60 m was sufficient for fatty acid and cis and trans isomers of fatty acids. The following conditions were employed for the separation of FAMEs of C4 to C26: Injector temperature: 250 °C; detector temperature: 250 °C; oven temperature: 165 °C (8 min) to 210 °C at 2 °C/min; carrier gas hydrogen: column head pressure 179 kPa; total flow: 154.0 mL/min; split ratio: 1:100 and injection volume: 1 μl.

| **a** | **b** |
| --- | --- |
|  |  |

**Fig. S1** Eadie-Hofstee plot for the determination of the apparent Michaelis-Menten constant Km of (a) the covalently immobilized enzyme on the electrode surface using the multi-step approach for sensor functionalization and (b) the entrapped enzyme on the CB/CS matrix using the one-step approach for surface functionalization. Each point is the mean value of three measurements ± SD.
